# Novel insights into habitat suitability for Amazonian freshwater mussels linked with hydraulic and landscape drivers

**DOI:** 10.1002/ece3.7947

**Published:** 2021-08-13

**Authors:** Diego Simeone, Claudia Helena Tagliaro, Colin Robert Beasley

**Affiliations:** ^1^ Laboratório de Conservação da Biodiversidade e das Águas Instituto de Estudos Costeiros Universidade Federal do Pará Bragança Brazil

**Keywords:** fluvial system, random forest, riparian buffer, riverbed stability, Unionida bivalves

## Abstract

Novel insights into habitat suitability for two Unionida freshwater mussels, *Castalia ambigua* Lamarck, 1819 (Hyriidae) and *Anodontites elongatus* (Swainson, 1823) (Mycetopodidae), are presented on the basis of hydraulic variables linked with the riverbed in six 500‐m reaches in an eastern Amazonian river basin. Within the reaches, there was strong habitat heterogeneity in hydrodynamics and substrate composition. In addition, we investigated stressors based on landscape modification that are associated with declines in mussel density. We measured hydraulic variables for each 500‐m reach, and landscape stressors at two spatial scales (subcatchment and riparian buffer forest). We used the Random Forest algorithm, a tree‐based model, to predict the hydraulic variables linked with habitat suitability for mussels, and to predict which landscape stressors were most associated with mussel density declines. Both mussel species were linked with low substrate heterogeneity and greater riverbed stability (low Froude and Reynolds numbers), especially at high flow (low stream power). Different sediment grain size preferences were observed between mussel species: *Castalia ambigua* was associated with medium sand and *Anodontites elongatus* with medium and fine sand. Declines in mussel density were associated with modifications linked to urbanization at small scales (riparian buffer forest), especially with percent of and distance from rural settlements, distance to the nearest street, and road density. In summary, the high variance explained in both hydraulic and landscape models indicated high predictive power, suggesting that our findings may be extrapolated and used as a baseline to test hypotheses of habitat suitability in other Amazonian rivers for *Castalia ambigua* and *Anodontites elongatus* and also for other freshwater mussel species. Our results highlight the urgent need for aquatic habitat conservation to maintain sheltered habitats during high flow as well as mitigate the effects of landscape modifications at the riparian buffer scale, both of which are important for maintaining dense mussel populations and habitat quality.

## Graphical abstract

Random Forest algorithms with complex hydraulic variables and landscape stressors predicted that Amazonian mussels are associated with riverbed stability at high and low flows and mussel density is higher with low substrate heterogeneity and hydrodynamic energy. Local riparian buffer modification by urbanization leads to mussel density declines, and there is an urgent need for landscape and riverscape management and conservation for maintenance of mussel populations and habitat quality.

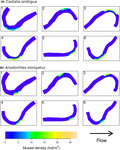

## 1 INTRODUCTION

Rivers are complex ecosystems that vary in terms of morphology and hydrodynamics (Gordon et al., 2004). Flow is both an important driver and descriptor of habitat heterogeneity (Strayer et al., 2006) and species distribution (Silva & Yalin, 2017). Freshwater mussels (hereafter mussels) constitute most of the invertebrate biomass in rivers, living partially or completely buried in the sediment (Watters, 1994), and are strongly affected by changes in flow conditions (Goodding et al., 2019; Stoeckl & Geist, 2016). Many studies have been carried out in temperate rivers to identify mussel habitat (Morales et al., 2006; Smit & Kaeser, 2016; Zigler et al., 2008) and understand their distribution (Goodding et al., 2019; Hegeman et al., 2014; Maio & Corkum, 1995; Steuer et al., 2008). *Simple* hydraulic variables (e.g., current velocity and depth) produced weak predictions for the identification of these habitats (Hardison & Layzer, 2001) because they did not reflect the real influence of flow on the riverbed (Allen & Vaughn, 2010). However, *complex* hydraulic variables (e.g., shear stress, Froude and Reynolds numbers), related to near‐bed flow conditions, were more robust for predicting habitat suitability (Strayer, 1999).

Identification of suitable habitats for mussels is important for aquatic conservation because they may be found in a diverse array of habitats (Dobler et al., 2019; Smit & Kaeser, 2016). For example, mussels may occur in gravely or sandy river substrates (Allen & Vaughn, 2010; Steuer et al., 2008), whereas others may aggregate among large cobbles (Gangloff & Feminella, 2007) and similar flow refuges, which are sheltered and remain stable in periods of high flow (Morales et al., 2006). Furthermore, mussel distributions may occur over large spatial scales via dispersal of their larval stages on host fish, or over small spatial scales in the reaches where they settle and aggregate (Atkinson et al., 2012; Hornbach et al., 2019; Poole & Downing, 2004). Mussel populations have been declining drastically over decades for diverse reasons (Böhm et al., 2020), resulting in lower population sizes and local extinctions (Hamstead et al., 2019; Shea et al., 2012). Of particular relevance to the present study are landscape modifications for agriculture (Cao et al., 2013; Daniel & Brown, 2013; Daniel et al., 2018; Zieritz et al., 2016) and river channel alterations that decrease mussel/aquatic habitat heterogeneity (Arbuckle & Downing, 2002).

In South America, precise information on suitable mussel habitat is lacking, where substrate characteristics (e.g., grain size) or proportions of sediment organic matter have sometimes been reported (Mansur & Pereira, 2006 and references therein), especially for wetlands (Santos et al., 2020 and references therein). Shear velocity was used to describe the distribution of mussels and associated macroinvertebrates in meanders in an eastern Amazon River (Simeone et al., 2018), but not for other hydraulic habitats, such as backwaters and straight reaches, nor were substrate characteristics described in detail. Furthermore, there are no studies from the Amazon that use landscape variables to measure the effect of these stressors on mussel density. This is of great concern because the Amazon landscape has been modified by conversion of forest to pasture and agro‐industrial plantations (Marengo et al., 2018) and by ongoing infrastructural development involving mining, dams, ports, and roads (Walker et al., 2019).

This is the first study to use complex hydraulic and landscape variables to predict habitat suitability for two mussel species (*Castalia ambigua* and *Anodontites elongatus*), which are widely distributed in the Amazon (Pereira et al., 2014). Modeling based on both these types of variables is a powerful tool for extrapolating predictions for mussel suitable habitats in unsampled rivers (Cao et al., 2015). However, interactions between variables (multicollinearity) are common in environmental data which may violate the key assumptions of more traditional statistical models (Breiman, 2001). Machine‐learning methods (e.g., Random Forests) may overcome many of these limitations, especial removing over‐fitting, and may generate more robust predictions (Liaw & Wiener, 2002).

Different to North America and Europe, there is little or no monitoring of the mussel fauna by environmental agencies in Brazil, especially in the Amazon, hindering a complete inventory of the species and their distributions. In addition, in large Amazonian rivers, mussel beds are distributed over a wide spatial scale (Pereira et al., 2014), which increases transit time between sampling locations (Smit & Kaeser, 2016) and reduces sampling effort. Our fieldwork was carried out in a single river basin in the eastern Amazon, in order to better control for variation in habitat conditions. We aimed to identify suitable habitats for *Castalia ambigua* and *Anodontites elongatus*, comparing periods of high and low flows, identify the hydraulic variables most closely linked with habitat suitability, and the landscape stressors that are associated with declines in mussel density. We hypothesized that *Castalia ambigua* and *Anodontites elongatus* would be associated with areas that are stable in both periods of high and low flows (Hardison & Layzer, 2001; Randklev et al., 2019) and with high substrate heterogeneity (Garcia et al., 2012; Strayer et al., 2006). We also hypothesized that declines in mussel density would be associated with greater landscape modification, caused mainly by increased urbanization (Brown et al., 2010; Gillis et al., 2017).

## 2 METHODS

### 2.1 Study area and mussel species

**FIGURE 1:**
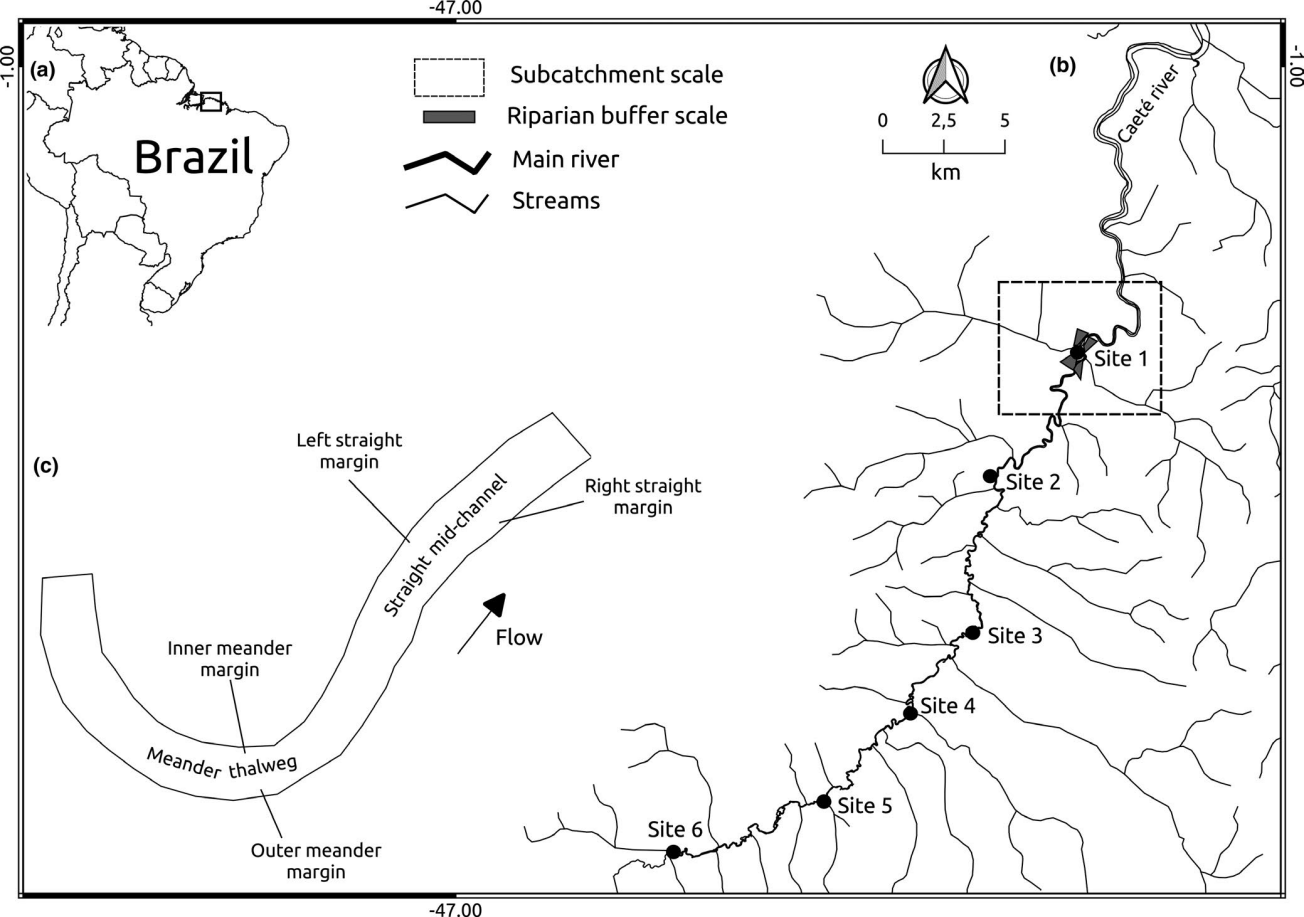
Study area in northern Brazil (a), with location of the six sites in the middle course of the Caeté River, approximately 30 km upstream of the city of Bragança, Pará, Brazil, and an example of the subcatchment and buffer scales in site 1 used for landscape modeling (b). Example of mesohabitat locations in a 500‐m reach (c)

The present study was carried out in the middle course of the Caeté River, a morphologically unaltered alluvial lowland river, approximately 150 km long with a sixth‐order basin, located in northeastern Pará state, in the eastern Brazilian Amazon (Figure 1). There is marked seasonality in river hydrology, which is a feature of Amazonian rivers (Junk, 1997), with an average discharge (± *SD*) of 48.3 ± 11.5 m^3^/s in the rainy season and 8.4 ± 2.9 m^3^/s in the dry season (Simeone et al., 2018). The Caeté has a predominantly meandering morphology, with regular meanders in the upper course, and, in the middle course, irregular and tortuous meanders with the formation of central sandbanks in the thalweg (Simeone et al., 2018). The landscape consists of secondary forest floodplain, with small scattered human settlements, subsisting mainly by fishing and family‐based farming. Since large human settlements are scarce along the Caeté River, fluvial habitats are in relatively natural conditions, and there have been no artificial modifications to the channel along the course of the river.

Two mussel species occur in the Caeté River, *Anodontites elongatus* and *Castalia ambigua*, of which the latter dominates the beds (Simeone et al., 2021a). *Anodontites elongatus* is distributed from the Amazon‐Orinoco ecoregion to the southern Uruguay River. This species may occur together with *Castalia ambigua*, sharing the same habitat in marginal areas of rivers and lakes (Pereira et al., 2014), mainly associated with fine sand (Mansur & Valer, 1992). *Castalia ambigua* is distributed in the Amazon‐Orinoco ecoregion (Pereira et al., 2014), in lakes and marginal areas of lowland rivers, with loosely compacted sandy substrate (Mansur & Valer, 1992).

### 2.2 Sampling design

During low flow, between October and December 2018, we selected six sites known to have mussels (Figure 1b). We established one 500‐m reach per site covering all available habitats with different sediment characteristics and hydrodynamics (e.g., meanders, backwaters, and straight stretches). We classified mesohabitats in each reach according to substrate characteristics and water flow for a better habitat description (Silva & Yalin, 2017; Smit & Kaeser, 2016). We identified six distinct mesohabitats (Figure 1c; Table 1) that differed in terms of riverbed stability, substrate classification and grain size, and water flow (Table 1). Along each mesohabitat, we established two equidistant 100‐m transects. We placed twenty‐five 1‐m² plots along each transect, 300 plots per reach, by selecting a random position in such a way as to avoid clumping of plots and spatial autocorrelation, which can inflate the significance of statistical models (Cao et al., 2013, 2015). Geographic coordinates of each plot were recorded using a global positioning system (GPS) to build point patterns.

**TABLE 1:** Summary of riverbed stability, substrate composition and size, and mean ± standard deviation of the water speed at high and low flows in the mesohabitats identified at six 500‐m reaches in the Caeté River, Bragança, Pará, Brazil. Sediment classification and grain size follow the Wentworth scale in Gordon et al. (2004)

Mesohabitats	Riverbed stability	Sediment classification	Grain size range (mm)	Water speed at high flow (m/s)	Water speed at low flow (m/s)
Outer meander margin	Stable	Fine to medium sand	0.125–0.5	0.7 ± 0.2	0.4 ± 0.1
Meander thalweg	Unstable	Coarse sand to gravel	0.5–2	1.2 ± 0.5	0.9 ± 0.3
Inner meander margin	Stable	Silt to very fine sand	0.03–0.125	0.5 ± 0.1	0.1 ± 0.1
Left straight margin	Unstable	Medium to coarse sand	0.25–1	1.1 ± 0.3	0.6 ± 0.2
Straight mid‐channel	Unstable	Gravel	1–2	1.7 ± 0.8	1.3 ± 0.5
Right straight margin	Unstable	Coarse sand	0.5–1	1.2 ± 0.3	0.7 ± 0.5

### 2.3 Complex hydraulic variables

During low flow, current velocity (m/s), measured at 1 cm above the river bed using a digital Flowatch meter (precision 0.01 m/s), and depth (m), measured using a metric stick, were obtained at the center of each plot and used to calculate a set of nine hydraulic variables using the formulas in Table 2. We used a Shield's parameter (*θ*_c_) of 0.035 because substrates at our sites consisted of mostly sand with a generally random grain arrangement (Gordon et al., 2004). We collected sediment at the center of each plot, to determine substrate composition. In the laboratory, 100 g of the dried sediment (48 hr at 60°C) was passed through a series of six geological sieves (2, 1, 0.5, 0.25, 0.125, and 0.063 mm), and each fraction weighed (precision 0.001 g).

**TABLE 2:** Summary of complex hydraulic variables estimated at six sites in the Caeté River, Bragança, Pará, Brazil. *D*_x_ = substrate particle size (mm) at an *x%* of the sample, *d* = water depth (m), *ϕ* = phi unit of substrate size (*ϕ* = −log_2_ *D* [mm]), *ϕx* = substrate particle size (ϕ) at an *x%* of the sample, *U* = mean current velocity (m/s), g = acceleration of gravity (9.8 m/s), υ = kinematic viscosity of water (0.0000176 m^2^/s), *μ* = dynamic viscosity of water (0.000173 Pa.s), *ρ* = density of water (996 kg/m^3^ for an average temperature of 28°C), *ρ*_s_ = density of substrate (273 kg/m^3^), *dv*/*dy* = velocity gradient (rate of change in velocity [*dv* in m/s], with distance [dy in m] which was estimated to a depth of 0.01 m), *θ*_c_ = Shield's parameter (0.035). Formulas in Gordon et al. (2004) and Silva and Yalin (2017)

Complex hydraulic variables	Formula	Description
Substrate variables		
*D* (mm)	$\frac{\left(D_{16}+D_{50}+D_{84}\right)}{3}$	Mean particle size
Sorting index (*S*_o,_ unitless)	$\frac{\left(\phi 84‐\phi 16\right)}{2}$	Substrate heterogeneity
Bed roughness (*k*_s_, mm)	2 × *D*_50_	Topographical variation of river bed
Hydraulic variables		
Froude number (Fr, unitless)	$\sqrt{\frac{U^{2}}{\mathrm{gd}}}$	Inertia ratio of gravitational force
Reynolds number (Re, unitless)	$\frac{\mathrm{Ud}}{\upsilon }$	Turbulence of free flow
Boundary Reynolds number (Re_*_, unitless)	$\frac{Vk_{s}}{\upsilon }$	Near‐bed turbulence
Shear velocity (*V*, m/s)	$\sqrt{\frac{\tau }{\rho }}$	Friction velocity
Shear stress (*τ*, N/m^2^)	$\mu \frac{d\nu }{\mathrm{dy}}$	Friction force on substrate
Critical shear stress (*τ*_c_, N/m^2^)	$\theta_{c}gD_{50}\left(\rho_{s}‐\rho \right)$	Shear stress required to initiate substrate motion

Studies that used hydraulic variables to predict mussel distribution have usually sampled in periods of high and low flows (Allen & Vaughn, 2010; Randklev et al., 2019). We could not access mussel beds at high flow due to the higher water level and current velocity. Therefore, between March and April 2019, measurements were taken from a boat secured by a cable. We randomly selected fifteen plots, from those recorded at low flow, along each transect at each mesohabitat (180 plots per reach). Afterward, we measured the current velocity (m/s) at 1 cm above the river bed using a digital Flowatch meter (precision 0.01 m/s) fixed to a weight of 5 kg and depth (m) using a cable marked every 0.5 m. With these variables, we calculated the stream power (*w*_a_, N/m s) that describes substrate stability and sediment transport in the riverbed, identifying sheltered habitats used for mussels in the period of high flow, using the formula in Gordon et al. (2004):

$$w_{a}=\tau U,$$

where *τ* is the shear stress at the riverbed (N/m^2^), and *U* is the mean current velocity (m/s).

### 2.4 Mussel sampling

We sampled for mussels in each plot as the last step of the fieldwork to avoid bias during measurements of hydraulic variables. We sampled mussels by manually excavating the sediment to a depth of approximately 15 cm and by conducting semi‐quantitative timed searches, which provide better spatial coverage and are useful for rare mussel species (Allen & Vaughn, 2010). Searches were carried out by a 2‐ to 3‐member crew until all mussels present at each plot were removed. All mussels were identified to species, their density quantified, and returned to the sediment. We did not include dead mussels because these could have been transported during periods of high flow.

### 2.5 Landscape variables

The influence of landscape on mussel density was analyzed at different spatial scales as suggested by Hopkins (2009). Two spatial scales were used as follows: subcatchment scale (drainage area of 5 km^2^ associated with each sampling site) and riparian buffer scale (200 m riparian buffer extending 1 km along the river margin from each sampling site). We extracted a set of thirteen landscape variables (Table 3) including information on roads, urban settlements, and riparian vegetation from both sides of the river (*n* = 12 at the site scale) using layers from OpenStreetMap (https://www.openstreetmap.org) available via the OpenLayers plugin in QGIS 3.12 (QGIS Development Team, 2020), and land‐use information from Instituto Brasileiro de Geografia e Estatística (IBGE) available at https://www.ibge.gov.br/geociencias/informacoes‐ambientais/cobertura‐e‐uso‐da‐terra. Landscape variables were classified according to three main groups of stressors that may affect mussel density (Cao et al., 2015; Gillis et al., 2017; Hopkins, 2009): riparian forest cover, land use, and urbanization (detailed description in Table 3).

**TABLE 3:** Summary of landscape variables measured at two different spatial scales (subcatchment and riparian buffer forest) at six sites in the Caeté River, Bragança, Pará, Brazil

Landscape variables	Description
*Subcatchment scale*	
Riparian forest cover	
R_Area	Riparian zone area (km^2^)
Land use	
N_Forest	Number of forest patches per km^2^
N_Agriculture	Number of agricultural patches per km^2^
N_Pasture	Number of pasture patches per km^2^
Urbanization	
N_RuralSet	Number of rural settlements patches per km^2^
*Riparian buffer scale*	
Riparian forest cover	
Can_Cover	Canopy cover (%)
Land use	
P_Forest	Percent of forest in a 200 m riparian zone (%)
P_Agriculture	Percent of agriculture in a 200 m riparian zone (%)
P_Pasture	Percent of pasture in a 200 m riparian zone (%)
Urbanization	
D_RuralSet	Distance to the nearest rural settlement (m) in a 200 m riparian zone
D_Street	Distance to the nearest street (m) in a 200 m riparian zone
P_RuralSet	Percent of rural settlements in a 200 m riparian zone (%)
D_Road	Road density (average number of road in a 200 m riparian zone)

### 2.6 Random forest modeling

We used Random Forest (RF) regression in the *randomForest* package (Liaw & Wiener, 2002) in GNU R 4.0.1 (R Core Team, 2020) to build two types of mussel models. Firstly, we modeled the density of *Castalia ambigua* and *Anodontites elongatus* to predict suitable habitats using complex hydraulic variables. Here, we used a Repeated Measures Random Forest (RMRF) to take into account possible nonindependence of the randomly selected replicates (plots) within each site and potential pseudoreplication (Calhoun et al., 2021). We used the replicates (plots) as the random effect. This model aimed to identify hydraulic conditions (Table 2) linked with habitat suitability for mussels. Secondly, we used a traditional RF for modeling the density of *Castalia ambigua* and *Anodontites elongatus* using landscape variables (Table 3) to predict which stressors were associated with declines in mussel density among sites. In the Caeté River, harvesting drastically reduced mussel density between the years 2015 and 2017 at sites 1 and 2. However, mussel beds at the other sites are well conserved and have high mussel densities (Simeone et al., 2021a).

Initially, we used log (*x* + 1) transformation on the raw mussel density data to focus over the lower range of individuals which is more ecologically meaningful than over the higher range (Bocard et al., 2011). We overtrained our model by selecting a subsample from our whole dataset by bootstrapping. Afterward, we ran the training model progressively, increasing the numbers of predictors used for group splitting (*mtry* function in the *randomForest* package; Liaw & Wiener, 2002). We tested the following settings: mtry = 1 to 9 for hydraulic variables and mtry = 1 to 13 for landscape variables. We reran these models five times at each level of mtry using different random seeds, as follows: *ntree* function = 200, 300, 400, 500, 600, 800, and 1,000. Observations which were not included in the bootstrap subsample were defined as out‐of‐bag (oob) samples and were used to create an oob estimation of the generalized error in the model (Breiman, 2001). The optimum number of mtry to be used in final models was selected from the forest with the smallest generalized error (Breiman, 2001; Liaw & Wiener, 2002).

For our final RMRF and RF models, we chose those with the highest variance explained (pseudo‐*R* ^2^ values) for the oob samples. Random Forests compute an importance value for each predictor, which gives information about the relationship between this predictor and the response variable (Breiman, 2001). Thus, we used the increases in mean standard error (*MSE*) to evaluate this relationship (Liaw & Wiener, 2002). Higher *MSE* values indicate greater importance of predictor variables. Finally, the relationship between hydraulic and landscape predictors and the response of *Castalia ambigua* and *Anodontites elongatus* were described using partial dependence plots in the *randomForest* package (Liaw & Wiener, 2002).

### 2.7 Mussel mapping distribution

Point patterns obtained to show the location of suitable habitats for mussels were plotted by spatial smoothing densities of *Castalia ambigua* and *Anodontites elongatus* with the *spatstat* package (Baddeley & Turner, 2005) in GNU R 4.0.1 (R Core Team, 2020). Shapefiles used as the plotting window were obtained from the OpenLayers plugin in QGIS 3.12 (QGIS Development Team, 2020). Subsequently, we found the minimum bounding box size to specify the dimensions of each plotting window and obtain sufficiently detailed maps. We used mussel density as marks to create the point pattern object in the *ppp* function. Afterward, we applied the Kernel smoothed intensity estimator in the *Smooth.ppp* function to plot the densities of *Castalia ambigua* and *Anodontites elongatus*. We selected an appropriate smoothing bandwidth for the *sigma* parameter in the plotting function (Baddeley et al., 2016), using the cross‐validation function *bw.diggle* to minimize the mean squared error criterion (Diggle, 2003). Finally, we carried out the Hopkins–Skellam test, which uses the nearest‐neighbor distance, to calculate the pattern distribution of the randomly chosen mussel density plots (Hopkins, 1954). A Hopkins–Skellam value <1 suggests clustering and >1 regularity in the distribution of the mussel density plots.

## 3 RESULTS

### 3.1 Model training

The best RMRF hydraulic model was obtained with mtry = 4 for *Castalia ambigua* and mtry = 2 for *Anodontites elongatus*. Performance was not substantially enhanced after ntree = 600, so we selected this number as the maximum number of trees for the final model. The oob generalized error was 0.2 for *Castalia ambigua* and 0.05 for *Anodontites elongatus*. For the RF landscape model, the best model was reached with mtry = 3 for both *Castalia ambigua* and *Anodontites elongatus*. Performance was not substantially enhanced after ntree = 600, so we selected this number as the maximum number of trees for the final model. The oob generalized error was 0.04 for *Castalia ambigua* and 0.004 for *Anodontites elongatus*.

### 3.2 Habitat suitability for mussels based on complex hydraulic variables

**FIGURE 2:**
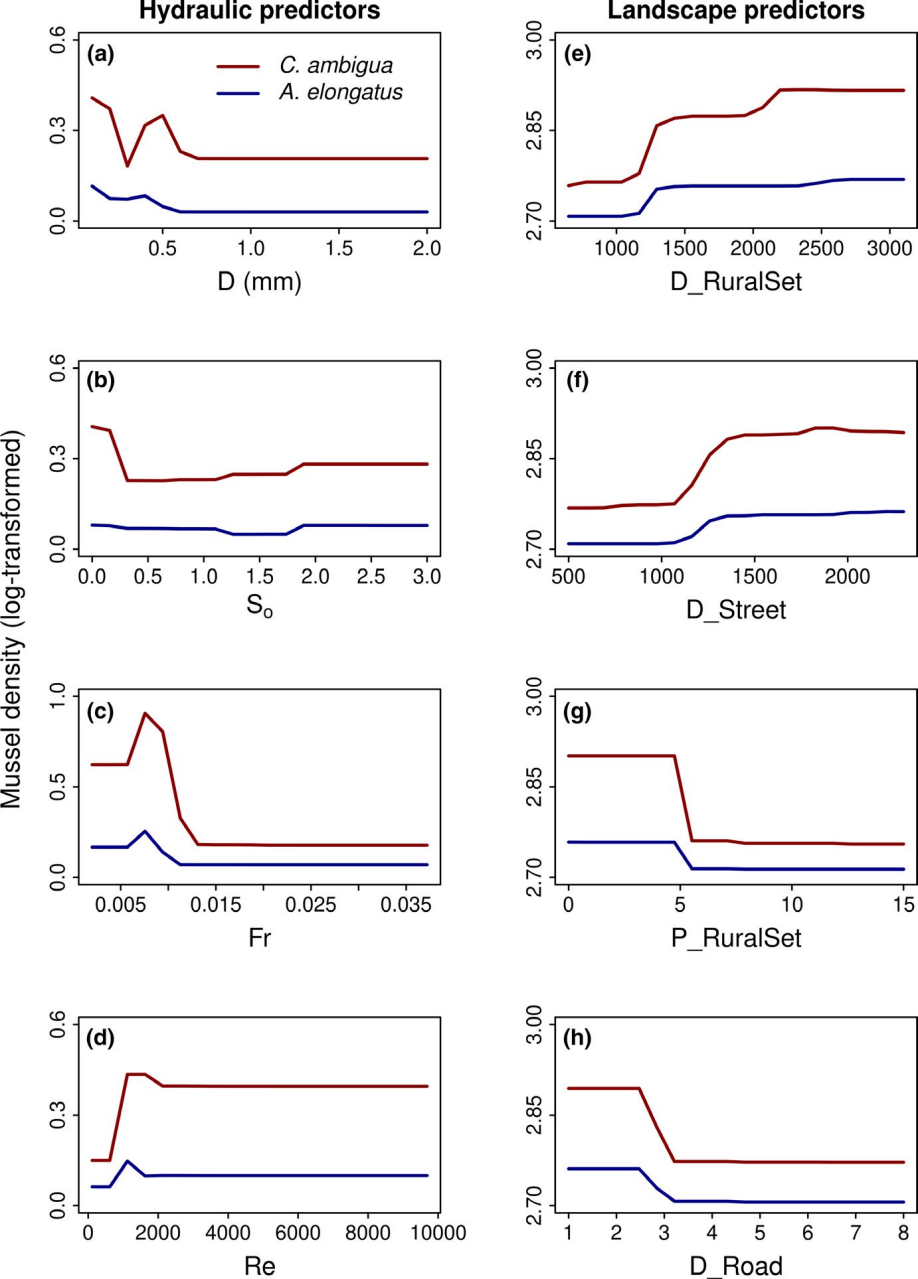
Partial dependence plots based on Random Forest regression, showing the relationship of the four hydraulic and landscape key predictors, with densities of *Castalia ambigua* and *Anodontites elongatus*, sampled at six sites in the middle course of the Caeté River, Bragança, Pará, Brazil. Hydraulic predictors were mean particle size (*D*; mm) [a], sorting index (*S*_o_; unitless) [b], Froude number (Fr; unitless) [c], and Reynolds number (Re; unitless) [d]. Landscape predictors were distance to the nearest rural settlement in a 200‐m riparian zone (D_RuralSet) [e], distance to the nearest street in a 200‐m riparian zone (D_Street) [f], percent of rural settlements in a 200 m riparian zone (P_RuralSet) [g], and road density (average number of roads in a 200‐m riparian zone; D_Road) [h]

The final RMRF hydraulic model to predict suitable habitats for mussels explained (pseudo‐*R* ^2^) 70.5% of the total variance for *Castalia ambigua* and 49.7% for *Anodontites elongatus*. The high variance explained and low oob generalized error indicated high predictive power of our model. Both species had the same subset of important predictors (first four predictors ranked in Table 4, upper section). Suitable habitats for *Castalia ambigua* were linked with medium sand and for *Anodontites elongatus* with medium and fine sand, with mean particle sizes of 0.3–0.5 mm and 0.2–0.4 mm, respectively (Figure 2a). In addition, both mussel species were linked with a substrate sorting index ranging from 0.1 (very well sorted) to 0.9 (moderately sorted) (Figure 2b), Froude number around 0.005–0.075 (Figure 2c), and Reynolds number around 500–1,800 for *Castalia ambigua* and 500–1,000 for *Anodontites elongatus* (Figure 2d). Both mussel species were absent below and above these thresholds.

**TABLE 4:** Ranking of the importance of hydraulic and landscape predictors for density of *Castalia ambigua* and *Anodontites elongatus* based on % increase in mean standard error (*MSE*) sampled at six sites in the Caeté River, Bragança, Pará, Brazil

Rank	*Castalia ambigua*		*Anodontites elongatus*	
	Hydraulic predictors	% increase in *MSE*	Hydraulic predictors	% increase in *MSE*
1	*D* (mm)	47.0	Fr (unitless)	22.5
2	Fr (unitless)	37.6	*D* (mm)	22.4
3	*S*_o_ (unitless)	36.7	*S*_o_ (unitless)	22.4
4	Re (unitless)	30.1	Re (unitless)	22.3
5	Re_*_ (unitless)	25.4	*τ* (N/m^2^)	17.1
6	*τ* (N/m^2^)	22.5	*V* (m/s)	16.8
7	*V* (m/s)	22.3	Re_*_ (unitless)	15.9
8	*τ*_c_ (N/m^2^)	17.1	*τ*_c_ (N/m^2^)	14.8
9	*k*_s_ (mm)	15.4	*k*_s_ (mm)	14.1

Rank	Landscape predictors	% increase in *MSE*	Landscape predictors	% increase in *MSE*
1	D_RuralSet	9.1	D_RuralSet	9.6
2	P_RuralSet	8.9	P_RuralSet	8.8
3	D_Road	8.5	D_Street	8.7
4	D_Street	8.4	D_Road	7.1
5	R_Area	5.4	R_Area	6.9
6	P_Forest	2.6	Can_Cover	1.9
7	N_Agriculture	1.0	P_Forest	1.9
8	N_Pasture	0.9	P_Pasture	1.2
9	P_Pasture	0.7	N_RuralSet	0.0
10	N_RuralSet	0.0	N_Forest	−0.8
11	P_Agriculture	−0.7	N_Agriculture	−1.0
12	N_Forest	−0.9	N_Pasture	−1.1
13	Can_Cover	−1.4	P_Agriculture	−1.9

**FIGURE 3:**
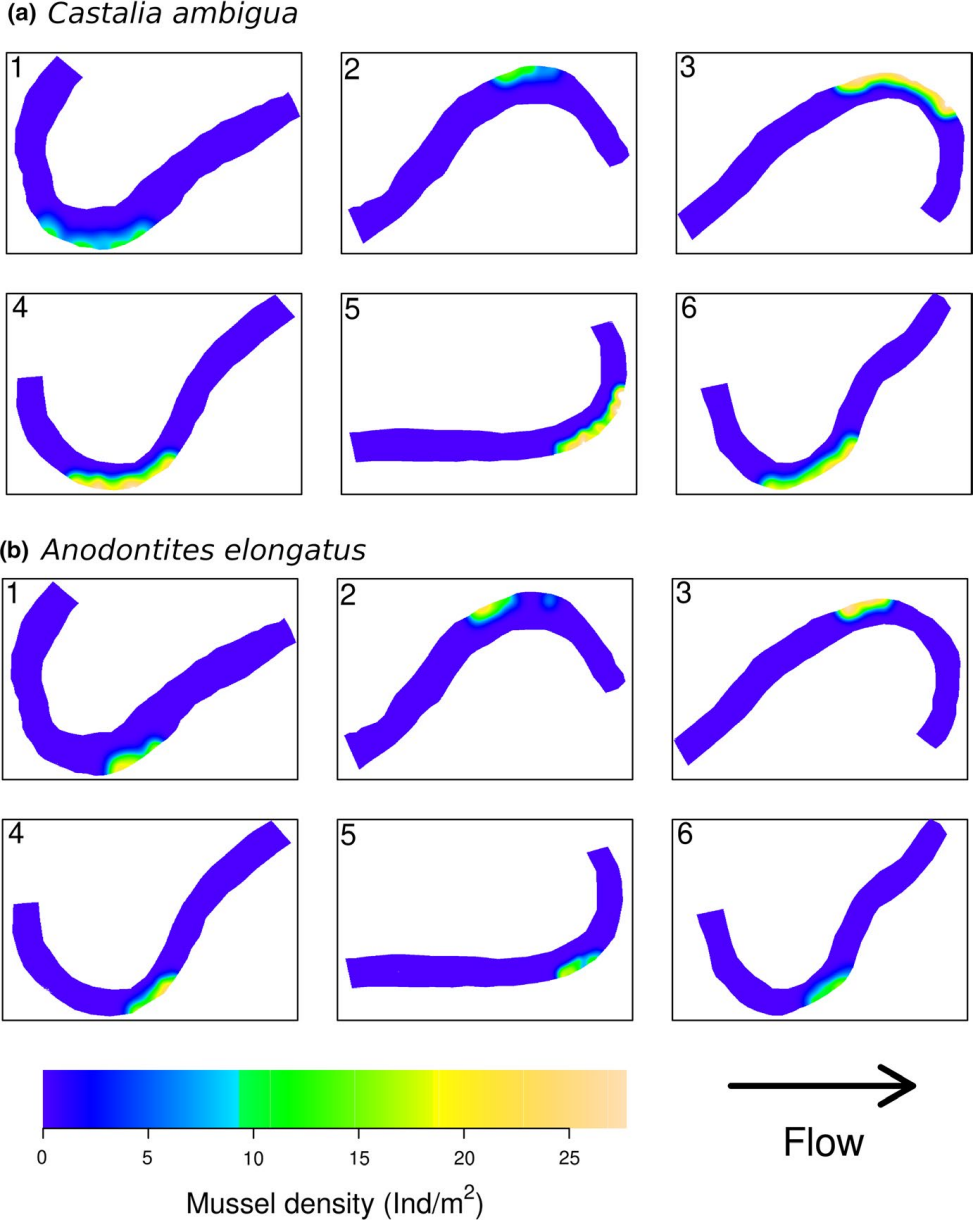
Smoothed density maps of the density (Ind/m^2^) of *Castalia ambigua* (a) and *Anodontites elongatus* (b), sampled at six 500‐m reaches in the Caeté River, Bragança, Pará, Brazil. Density is indicated by a color gradient. Arrow indicates direction of water flow

**FIGURE A1:**
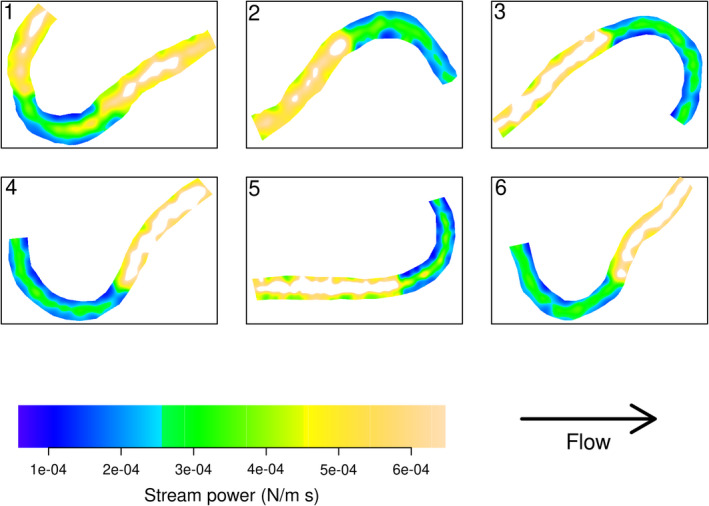
Smoothed maps of stream power (N/m s) values at high flow, sampled at six 500‐m reaches in the Caeté river, Bragança, Pará, Brazil. Stream power is indicated by the color gradient below. Arrow indicates direction of water flow

Smoothed density maps did show a consistent pattern for the location of *Castalia ambigua* (Figure 3a) and *Anodontites elongatus* (Figure 3b) for all sites. Overall, *Castalia ambigua* occurred along the meander outer bend mesohabitat, with a discrete clustered occurrence at a high density (Hopkins–Skellam test A = 0.91, *p* = .01; Figure 3a). On the other hand, *Anodontites elongatus* had a more restricted clustered occurrence in the meander outer bend (Hopkins–Skellam test A = 0.2, *p* < .001; Figure 3b) than that of *Castalia ambigua*. Both mussels species were absent in the straight and meander thalweg mesohabitats, which had higher hydrodynamics and greater sediment grain size, and in the meander inner bend that had low hydrodynamics and high silt deposition (Table A1). Furthermore, the meander outer bend showed higher riverbed stability at high flow with low values of stream power (Figure A1).

### 3.3 Decline in mussel density based on landscape variables

The final RF landscape model to predict the decline in mussel density explained (pseudo‐*R* ^2^) 69.3% of the total variance for *Castalia ambigua* and 86.8% for *Anodontites elongatus*. The high variance explained and low oob generalized error indicated high predictive power of our model. Both species had the same subset of important predictors (first four predictors ranked in Table 4, lower section). Density of *Castalia ambigua* and *Anodontites elongatus* increased with distance to the nearest rural settlement (D_RuralSet; Figure 2e) and distance to the nearest street (D_Street; Figure 2f), and decreased with percent of rural settlement (P_RuralSet; Figure 2g) and road density (D_Road; Figure 2h), especially for *Castalia ambigua* at sites 1 and 2 (Figure 3a), where rural settlements and road density were higher.

## 4 DISCUSSION

### 4.1 Habitat suitability for mussels linked with riverbed stability and low substrate heterogeneity

Our findings show that suitable habitats for *Castalia ambigua* and *Anodontites elongatus* are located in areas of low hydrodynamic energy, and low stream power at high flow. In temperate rivers, hydraulic conditions are more important for mussels than microhabitats *per se* (Allen & Vaughn, 2010; Box et al., 2002; Gangloff & Feminella, 2007). For example, variation in the hydrological regime may change flow conditions in microhabitats (Drew et al., 2018). This factor is critical for mussel distribution (Newton et al., 2008), because the number of suitable habitats decreases with increased flow (Hastie et al., 2000; Morales et al., 2006). In our study, meander outer bend mesohabitat supported mussel populations during flooding and had sufficient hydrodynamics at low flow to replenish water and avoid high deposition of silt. Mussels prefer habitats with enough stable substrate at high flow and with little or no deposition of silt (Hegeman et al., 2014), which is negatively associated with the presence of mussels (Quinlan et al., 2015). Similarly, in the meander inner bend of the Caeté River, where the riverbed was stable at high flow (low stream power), we observed high deposition of silt and the absence of mussels (present study and Simeone et al., 2018). Hydrodynamic conditions at the margins and the mid‐channel may vary in large‐ and medium‐sized rivers, especially in terms of flow and substrate type (Zigler et al., 2008). In Amazonian rivers, sandy banks may form in the mid‐channel (Junk, 1997). These habitats are also sheltered during flooding where deposits of medium and fine sand may build up (Junk, 1997). In temperate rivers, mussels are common in these habitats (Box et al., 2002; Christian et al., 2020; Morales et al., 2006), and since the variance explained in our model was high, we suggest that our findings may be extrapolated and used as a baseline to test hypotheses of habitat suitability in other Amazonian rivers for *Castalia ambigua* and *Anodontites elongatus* and also for other freshwater mussel species.

Riverbed stability was positively and strongly linked with densities of *Castalia ambigua* and *Anodontites elongatus*, supporting our first hypothesis and corroborating similar studies in temperate rivers (Hardison & Layzer, 2001; Randklev et al., 2019; Steuer et al., 2008). Habitats with more stable sediments, especially during high flow, may provide persistent shelter for mussels (Randklev et al., 2019; Wilson et al., 2011) and are thus important for juvenile mussel settlement (Morales et al., 2006). In our study, we did not differentiate mussels by age; however, we suggest that riverbed stability is an important driver for mussel colonization in Amazonian rivers. Low shear stress is responsible for providing hydraulic refuges for North American mussels (Gangloff & Feminella, 2007; Steuer et al., 2008; Strayer, 1999), since this variable is strongly associated with substrate stability, and high shear stress may limit habitat suitability for mussels (Stoeckl & Geist, 2016). In contrast, our study showed that riverbed stability was associated with Froude number, and especially Reynolds number, which differed between *Castalia ambigua* (500–1,800) and *Anodontites elongatus* (500–1,000). We suggest that *Castalia ambigua*, for having a more robust shell, supports habitats with relatively higher hydrodynamics, similar to those of sculptured mussel species in temperate rivers (Goodding et al., 2019). Froude and Reynolds numbers describe flow conditions near the river bed and are good predictors of habitat modification at small scales (Gordon et al., 2004). Hydraulic and substrate conditions at smaller scales may positively influence habitat suitability for mussels because flow near the riverbed may vary greatly over a few meters (Christian et al., 2020; Goodding et al., 2019). This was consistently observed in our study, as the mesohabitats we identified differed in terms of substrate stability and composition within short distances (~50 m).

In temperate rivers, substrate characteristics (e.g., grain size and sorting) appear not to be important in predictive models to describe suitable habitat for mussels (Cao et al., 2015; Maio & Corkum, 1995). In contrast, we observed that these predictors were strongly associated with the occurrence of suitable habitats for Amazonian mussels, which had distinct preferences for sediment grain size and sorting index. For example, *Castalia ambigua* was more associated with medium sand and very well sorted sediments. On the other hand, *Anodontites elongatus* preferred habitats with fine and medium sand and moderately sorted sediments, indicating the latter's preference for a slightly wider range of grain sizes. Differences in substrate preferences between species of *Castalia* and *Anodontites* were also observed in the Pantanal floodplain (Santos et al., 2020). Sorting regulates substrate heterogeneity, an important descriptor of species distribution (Quinlan et al., 2015; Strayer et al., 2006), since habitats with greater heterogeneity support higher species diversity and density (Garcia et al., 2012). In our study, mussels were not associated with very large grain size and high substrate heterogeneity, thus not entirely supporting our first hypothesis. In these habitats, sediment was mainly composed of coarse sand and gravel, which were negatively associated with the presence of mussels. We suggest that mussels prefer habitats with smaller grain size because it facilitates rapid burrowing into the riverbed, especially important during high flow (Allen & Vaughn, 2010; Goodding et al., 2019; Watters, 1994). The preference of both mussel species for different sediment types leads us to suggest a novel hypothesis that these habitats may influence Amazonian mussel shell morphology. For example, *Castalia ambigua,* which has a wider, rounded and robust shell, would burrow and move around more efficiently in habitats with medium sand, which are relatively loose and unconsolidated (Mansur & Valer, 1992). On the other hand, *Anodontites elongatus*, which has a thin and elongated shell, would burrow more easily in habitats with finer and more compacted sediments (Pereira et al., 2014). Similar patterns have been described for North American mussel species (Goodding et al., 2019; Watters, 1994) and should be more widely tested for these and other Amazonian mussel species.

### 4.2 Declines in mussel density associated with riparian buffer modification

Our study shows that declines in mussel density in the Caeté River were strongly associated with modifications in the landscape at the buffer scale, especially with predictors linked to riparian zone urbanization. These patterns were described by the same subset of predictors (% increase in *MSE*) for *Anodontites elongatus* and especially *Castalia ambigua*, which underwent greatest declines in density (Simeone et al., 2021a). Similar results were found by Cao et al. (2013) in North American rivers, where mussel density was negatively associated with road density and urban land use. In our study, percent of and distance from rural settlements, distance to the nearest street, and road density were especially associated with easy access to mussel beds. Mussels are harvested in eastern Amazonian rivers for the production of buttons (Beasley, 2001), and for medicinal use and food (Simeone et al., 2021a). Therefore, easy access to mussels reduces costs for harvesting (Smit & Kaeser, 2016) and increases exploitation of these populations (Beasley, 2001; Cao et al., 2013). The decrease in mussel density may be catastrophic for the aquatic ecosystem of the Caeté River (Simeone et al., 2021a), since they perform important ecological functions (Simeone et al., 2021b). For example, mussels deposit feces and pseudofeces that serve as food for other species and increase water quality through filtration (Simeone et al., 2021b; Vaughn & Hakenkamp, 2001). Mussel beds are better conserved and have higher densities in areas with lower urban development (Brown et al., 2010), which was consistently observed in our results. Natural riparian buffers maintain populations of mussels better than modified riparian areas (Atkinson et al., 2012; Poole & Downing, 2004; Zieritz et al., 2016). These observations highlight the importance of the conservation and maintenance of riparian zones in Amazonian rivers, since mussel density decreases with increased urban development around the river (Hopkins, 2009; Hornbach et al., 2018, 2019), and diversity of other associated macroinvertebrates decreases with mussel density (Simeone et al., 2021a).

Areas modified by pasture and agriculture also negatively affect mussel density (Daniel & Brown, 2013; Daniel et al., 2018; Poole & Downing, 2004). Soils disturbed by agriculture may modify river morphodynamics due to sediment inputs from increased bank erosion (Arbuckle & Downing, 2002; Zieritz et al., 2016). In contrast, land use associated with pasture and agriculture, both in terms of subcatchment and in terms of buffer scales, was not important stressors in our results. Our predictive model was generated using landscape data from a single river basin in eastern Amazon, where agriculture and pasture are not practiced on an intensive agro‐industrial scale (Instituto Brasileiro de Geografia e Estatística, 2013), so the effect of these stressors on mussel density decline may be greater in other rivers where large parts of the catchment continue to be modified by intensive agriculture, hydroelectric power plants, mining, infrastructure development, and deforestation for cattle ranching (Marengo et al., 2018; Walker et al., 2019).

Landscape modifications may alter and fragment the natural riverine habitat, resulting in declines and isolation of mussel populations (Böhm et al., 2020; Hamstead et al., 2019; Shea et al., 2012). Mussels are long‐lived and sedentary, and changes in the landscape may be quickly expressed at small spatial scales through riparian buffer alterations (Hopkins, 2009; Poole & Downing, 2004) and reflected over time at large spatial scales in the catchment, probably because natural buffers may mitigate catchment disturbances (Atkinson et al., 2012). Furthermore, mussels may show different patterns of longitudinal distribution, as was found for *Unio crassus* (wider distribution) and *Margaritifera margaritifera* (restricted distribution) in rivers of Bavaria, Germany (Dobler et al., 2019). Similar patterns were also found in our study for *Castalia ambigua* (wider longitudinal distribution) and *Anodontites elongatus* (restricted longitudinal distribution). Therefore, changes in Amazonian land‐ and riverscapes at different spatial scales may affect mussel density. For example, altered riparian buffer cover may decrease mussel density and the number of suitable habitats (Arbuckle & Downing, 2002), which was observed in our study and corroborated by evidence from temperate rivers (Poole & Downing, 2004). At a broader scale, other mechanisms may alter mussel populations, such as mussel dispersal and the host fish assemblage composition (Atkinson et al., 2012). However, there is no information about the host fishes used by Amazonian freshwater mussels. Therefore, future studies should include predictors associated with host fishes, mussel dispersal, and other land‐use patterns, such as mining and dams that may influence mussel populations in the Amazon. Management plans should include areas for conservation in both buffer and subcatchment scales since these are important for freshwater mussel distribution (Dobler et al., 2019).

## 5 CONCLUSION

Our findings provide novel insights into habitat suitability for Amazonian mussels linked with areas of greater stability (low Froude and Reynolds numbers), shelter during high flow (low stream power), and low substrate heterogeneity. Mussel species showed distinct preferences for sediment type: *Castalia ambigua* was associated with medium sand and *Anodontites elongatus* with medium and fine sand. Furthermore, mussel density was negatively associated with small riparian buffer scale landscape modification, linked to increased urbanization in the riparian zone. Our results show the importance of aquatic habitat conservation, especially of hydrodynamics and riparian habitat, which are important for maintaining dense mussel populations. Management plans should consider the diversity of habitats in terms of hydrodynamics and substrate type, which is important not only for mussels, but also for their host fish and, of course, aquatic diversity as a whole. Finally, urbanization plans should prioritize the conservation of riparian buffer habitats in order to maintain mussel populations and habitat quality.

## CONFLICT OF INTEREST

The authors declare no conflicts of interests.

## AUTHOR CONTRIBUTIONS

**Diego Simeone:** Conceptualization (equal); Formal analysis (equal); Writing‐original draft (lead); Writing‐review & editing (equal). **Claudia Helena Tagliaro:** Conceptualization (equal); Writing‐review & editing (equal). **Colin Robert Beasley:** Conceptualization (equal); Formal analysis (equal); Writing‐review & editing (equal).

## Data Availability

Data associated with this manuscript are available on Dryad (https://doi.org/10.5061/dryad.rfj6q57b2).

## Appendix

**TABLE A1:** Mean ± standard deviation of the hydraulic variables measured at the mesohabitats identified at six 500‐m reaches in the Caeté river, Bragança, Pará, Brazil. Hydraulic variables were: Mean particle size (*D*), sorting index (*S*_0_), bed roughness (*k*_s_), Froude number (Fr), Reynolds number (Re), boundary Reynolds number (Re_*_), shear velocity (*V*), shear stress (*τ*) and critical shear stress (*τ*_c_)

Hydraulic variables	Outer meander margin	Meander thalweg	Inner meander margin	Left straight margin	Straight mid‐channel	Right straight margin
*D* (mm)	0.4 ± 0.1	1.1 ± 0.2	0.2 ± 0.1	0.7 ± 0.2	1.7 ± 0.4	1.4 ± 0.2
*S*_0_	0.9 ± 0.4	1.1 ± 0.4	2.1 ± 0.8	0.9 ± 0.2	0.8 ± 0.4	1.1 ± 0.5
*k*_s_ (mm)	1.6 ± 0.4	3.4 ± 0.9	0.3 ± 0.1	3.2 ± 0.9	4.2 ± 1.4	4.7 ± 0.9
Fr	0.009 ± 0.0004	0.02 ± 0.001	0.003 ± 0.0003	0.01 ± 0.001	0.03 ± 0.002	0.01 ± 0.009
Re	1,289 ± 133	4,081 ± 537	331 ± 69	1555 ± 352	5,250 ± 657	1,725 ± 234
Re_*_	4,733 ± 1,499	11,083 ± 3,221	834 ± 386	11,930 ± 4,264	17,272 ± 6,587	16,369 ± 4,078
*V* (m/s)	0.0004 ± 0.0001	0.0005 ± 0.0001	0.0004 ± 0.0001	0.0006 ± 0.0001	0.0007 ± 0.0001	0.0006 ± 0.0001
*τ* (N/m^2^)	0.0002 ± 0.0001	0.0003 ± 0.0001	0.0002 ± 0.0001	0.0004 ± 0.0001	0.0005 ± 0.0001	0.0003 ± 0.0001
*τ*_c_ (N/m^2^)	−28,390 ± 8,121	−58,404 ± 15,615	−5,933 ± 2,206	−53,724 ± 16,995	−70,758 ± 25,055	−80,526 ± 16,539
